# Legislating for universal access to medicines: a rights-based cross-national comparison of UHC laws in 16 countries

**DOI:** 10.1093/heapol/czy101

**Published:** 2019-12-09

**Authors:** S Katrina Perehudoff, Nikita V Alexandrov, Hans V Hogerzeil

**Affiliations:** Department of Health Sciences, University Medical Center Groningen, University of Groningen, Hanzeplein 1, 9713, GZ, Groningen, The Netherlands

**Keywords:** Access, health insurance, legislation, human rights, essential drugs, government, accountability, health financing, user fees, vulnerable populations, equity

## Abstract

Universal health coverage (UHC) aims to ensure that all people have access to health services including essential medicines without risking financial hardship. Yet, in many low- and middle-income countries (LMICs) inadequate UHC fails to ensure universal access to medicines and protect the poor and vulnerable against catastrophic spending in the event of illness. A human rights approach to essential medicines in national UHC legislation could remedy these inequities. This study identifies and compares legal texts from national UHC legislation that promote universal access to medicines in the legislation of 16 mostly LMICs: Algeria, Chile, Colombia, Ghana, Indonesia, Jordan, Mexico, Morocco, Nigeria, Philippines, Rwanda, South Africa, Tanzania, Turkey, Tunisia and Uruguay. The assessment tool was developed based on WHO’s policy guidelines for essential medicines and international human rights law; it consists of 12 principles in three domains: legal rights and obligations, good governance, and technical implementation. Relevant legislation was identified, mapped, collected and independently assessed by multi-disciplinary, multi-lingual teams. Legal rights and State obligations toward medicines are frequently codified in UHC law, while most good governance principles are less common. Some technical implementation principles are frequently embedded in national UHC law (i.e. pooled user contributions and financial coverage for the vulnerable), while others are infrequent (i.e. sufficient government financing) to almost absent (i.e. seeking international assistance and cooperation). Generally, upper-middle and high-income countries tended to embed explicit rights and obligations with clear boundaries, and universal mechanisms for accountability and redress in domestic law while less affluent countries took different approaches. This research presents national law makers with both a checklist and a wish list for legal reform for access to medicines, as well as examples of legal texts. It may support goal 7 of the WHO Medicines & Health Products Strategic Programme 2016–30 to develop model legislation for medicines reimbursement.

**Key Messages**

- Health law makers in low- and middle-income countries (LMICs) lack the guidance and tools to write national legislation that promotes universal access to essential medicines through universal health coverage (UHC).
- This is the first study to assess the text of UHC laws for principles of access to medicines, based on WHO’s policies on essential medicines and international human rights law.
- Evidence from 16 mostly LMICs indicates that national laws often embed individual rights, government obligations, accountability and some technical principles (i.e. coverage for vulnerable groups), while other good governance and technical principles are infrequent.
- This article produces examples of legal text and an assessment tool, which is both a checklist for evaluating national law and policy, and a ‘wish list’ to guide legal reform.

## Introduction

Two billion people lack access to the medicines they need (Access to Medicines Foundation, 2015). Frequent public sector stock-outs, high medicines prices (especially in the private sector) and inadequate basic health insurance are major access barriers in low- and middle-income countries (LMICs) (Wirtz *et al.*, 2016). Faced with illness, households may incur catastrophic spending on medicines and endure the impoverishing consequences (Wirtz *et al.*, 2016). Universal access to essential medicines is therefore an important aspect of global development and a crucial component of universal health coverage (UHC), affirmed in the Sustainable Development Goal (SDG) 3 for Health. SDG Target 3.8 on UHC aims to ensure that all have access to health services including essential medicines without risking financial hardship (UN General Assembly, 2015). This target encompasses some aspects of the right to health, which offers a set of standards and principles for equitable and inclusive access to healthcare (Ooms *et al.*, 2014; Chapman, 2016a). Under international human rights law, governments have the ‘core obligation’ to provide essential medicines on a non-discriminatory basis and with attention for vulnerable groups (UN General Assembly, 1966b; UN CESCR 2000; Hogerzeil, 2006). Yet, SDG Target 3.8 neglects important aspects of a right to health approach, such as prioritized care and financial protection for the disadvantaged, and international cooperation and assistance for cost sharing with low-income countries (Ooms *et al.*, 2014; Chapman, 2016a).

According to WHO ‘[l]aw is a powerful tool for ensuring that the poor and vulnerable are not deprived of access to health care services and other resources for leading a healthy life.’ (Magnusson, 2017) A total of 165 States have ratified the International Covenant on Economic, Social and Cultural Rights (ICESCR), and therefore they bear the irrevocable duty to ensure their domestic law, policy, and practice protects and promotes these rights. On the path toward UHC over 70 countries have sought guidance from WHO concerning their domestic health reforms (WHO Consultative Group on Equity and Universal Health Coverage, 2014). WHO’s Medicines & Health Products Strategic Programme 2016–30 goal no. 7 is to develop model legislation for medicines reimbursement (WHO, 2017). Indeed, some reforms have been associated with increases in proxy indicators of medicines access such as their sale and use (Garabedian *et al.*, 2012; Nazzal *et al.*, 2016). However, UHC financing and access in other LMICs disproportionately benefits the rich compared with the poor (Asante *et al.*, 2016; Alshamsan *et al.*, 2017).

Human rights are increasingly acknowledged to help close the equity gap in UHC (Office of the United Nations High Commissioner for Human Rights and WHO, 2012; WHO Consultative Group on Equity and Universal Health Coverage, 2014). They can also guide an inclusive and accountable formulation of universal access to essential medicines in national law and policy (Hogerzeil, 2006; Ooms *et al.*, 2014; Sridhar *et al.*, 2015). In 2010, legal scholars in collaboration with WHO produced a pilot study of national legislation for access to medicines in four countries. Although this study was an important first step to collect and compare these national laws, it did not address UHC, likely reflecting the limited development of the concept at the time (Forzley *et al.*, 2010). In 2017 WHO published guidance to advise Member States on including the right to health in domestic law, including for UHC (Magnusson *et al.*, 2017). However, this guidance lacks a comprehensive approach to essential medicines as a health systems commodity and a component of UHC. Moreover, no practical assessment tool is available to aid lawmakers in analysing the strengths and weaknesses in national law for access to medicines.

Human rights principles in national legislation are an under-explored tool to enhance equitable medicines access through UHC in the existing literature. In existing scholarship, multiple, in-depth analyses of a single or small selection of countries study the evolution and impact of UHC reform on medicines and other health services (Carapinha *et al.*, 2011; Lagomarsino *et al.*, 2012; Atun *et al.*, 2013, 2015; Balabanova *et al.*, 2013). These studies focus on measures of health system function (i.e. pre-payment, pooling risk and purchasing) and of universal access (i.e. population coverage, services and direct costs to patients) (Hamilton *et al.*, 2016). However, these investigations neither use human rights law as the frame of reference, nor do they examine the content of national health legislation. Human rights principles (i.e. entitlements, State obligations, participation and accountability) create an enabling environment for patients to claim their rights and hold their governments accountable; therefore, these principles are also important aspects of legislation (Motta Ferraz, 2011; Chapman, Forman and Lamprea, 2017; Yamin and Maleche, 2017). Yet, previous studies of legal or policy interventions for medicines often source data from systematic literature reviews, websites of international organizations and/or key informants, rather than the laws or policies themselves (Vialle-Valentin *et al.*, 2008; Carapinha *et al.*, 2011; Gammie *et al.*, 2015; Hamilton *et al.*, 2016). This is a pragmatic albeit weak approach considering that no global repository of domestic health law exists and most health legislation is only available in the official national language(s) (Attaran *et al.*, 2012; Levels, Sluiter and Need, 2014).

To address these shortcomings, we develop and apply an assessment tool to UHC legislation in 16 mostly LMICs in order to identify legal texts that promote universal access to medicines. Our research presents examples of legal texts for domestic lawmakers and establishes a baseline of legal commitments to essential medicines in national law; it may support the WHO Medicines & Health Products Strategic Programme 2016–30 to develop model legislation for medicines reimbursement (WHO, 2017).

## Materials and methods

This is a cross-national study of UHC legislation for access to medicines from 16 mostly LMICs. It collects, describes and compares legal texts against an assessment tool with 12 principles for essential medicines and human rights. A detailed description of the methodology is in the Supplementary Appendix (https://dx.doi.org/10.6084/m9.figshare.6535106).

### Assessment tool

To analyse the content of UHC laws, we developed an assessment tool to identify 12 principles that are important from the perspective of WHO’s essential medicines policies (Hodgkin *et al.*, 2001; WHO, 2004, 2007, 2014; Bigdeli *et al.*, 2013) and international human rights law ( UN General Assembly, 1966a,b 2008, UN CESCR, 1991, 2000, 2006, 2007, 2016 ). (Selected international human rights law concerned States’ obligations toward social or health rights, the core obligation to provide essential medicines and/or rights related to good governance.) The principles are based on overlapping concepts related to access to medicines for vulnerable groups in the reference documents. The assessment tool was developed by two authors who shortlisted the relevant principles from source documents, independently piloted the short list on UHC laws to determine their applicability and adequacy, and revised the short list. Three rights to health and pharmaceutical policy experts (one author and two external reviewers) reviewed the short list to ensure the principles were sufficient and correctly defined.

We categorized the 12 principles in three domains (Table 1): legal rights and obligations (i.e. government’s commitments and duties), good governance (i.e. governance principles and processes) and technical implementation (i.e. policy measures to achieve government objectives). The domains correspond to the structure-process-outcome framework for monitoring and evaluating the realization of human rights (Office of the High Commissioner for Human Rights, 2012). We describe the principles and domains in our detailed methodology in the Supplementary Appendix (Perehudoff *et al.*, 2018). We hypothesize that recognizing all three domains in UHC legislation will generate more predictable, transparent and accountable rights and obligations, leading to greater responsiveness to the poor and vulnerable, equity, and sustainability.

**Table 1.** Assessment tool for access to essential medicines in national law and policy

Principles	Human rights principle	WHO essential medicines policy
**Legal rights and obligations**		
1. Right to health	Right to the highest attainable standard of health (UN General Assembly, 1966b; UN CESCR, 2000)	Human rights are a ‘value’ (Bigdeli *et al.*, 2013)
2. State obligation to provide essential medicines	Core obligation to provide essential medicines defined by WHO (UN General Assembly, 1966b; UN CESCR, 1991, 2000, 2008, 2016)	
**Good governance**		
3. Transparency	Transparency (UN General Assembly, 1966a,b; UN CESCR, 2000)	Includes information to assess service access and coverage, and publicly available price information for medicines. A component of good governance for medicines (Hodgkin *et al.*, 2001; WHO, 2007, 2014)
4. Participation and consultation	Participation (UN General Assembly, 1966a,b; UN CESCR, 2000)	Collaboration and accountability of all health systems actors, and stakeholder consultation is required. Referenced in good governance for medicines (Hodgkin *et al.*, 2001; WHO, 2007, 2014)
5. Monitoring and evaluation	Monitoring (UN General Assembly, 1966b; UN CESCR, 2000)	Achieved through explicit government commitment, indicator-based surveys and independent impact evaluation. A component of good governance for medicines (Hodgkin *et al.*, 2001; WHO, 2007, 2014)
6. Accountability and redress	Accountability (UN General Assembly, 1966a,b; UN CESCR, 2000)	Accountability of all health systems actors (WHO, 2007)
**Technical implementation**		
7. Selection of essential medicines	(Assured) quality of health services (of the AAAQ) (UN General Assembly, 1966b; UN CESCR, 2000)	Includes the essential drugs concept, procedures to define and update the national list(s) of essential drugs, explicit, evidence-based criteria that includes cost effectiveness and selection mechanisms (Hodgkin *et al.*, 2001; WHO, 2004)
	Duty to adopt appropriate legislative, administrative, budgetary and other measures to a maximum of its available resources Core obligation to provide essential medicines as defined by WHO (UN General Assembly, 1966b; UN CESCR, 1991, 2000, 2008, 2016)	
8. Government financing		Requires adequate funding and mobilizing all available public resources and increase funding for priority diseases, and the vulnerable (Hodgkin *et al.*, 2001; WHO, 2004, 2007)
9. Pool user contributions		Medicines reimbursement with user charges is a (temporary) financing option (WHO, 2004, 2007)
10. International assistance and technical cooperation	Duty to seek international assistance and technical cooperation (UN General Assembly, 1966b, 2008; UN CESCR, 2000, 2007)	Includes the possibility of using development loans for medicines financing (World Health Organization, 2004)
11. Efficient and cost-effective spending	Duty for the efficient use of available resources Duty to take appropriate steps to ensure that the private business sector is aware of, and consider the importance of, the right to health in pursuing their activities Duty to prevent unreasonably high costs for access to essential medicines from undermining the rights of large segments of the population to health Duty to seek low-cost policy options (UN General Assembly, 1966b, 2008; UN CESCR, 1991, 2000, 2008)	Includes the efficient use of resources and affordable pricing through: price control; a pricing policy for all medicines; competition through generic policies and substitution; good procurement practices; price negotiation and information; and TRIPS-compliant measures such as compulsory licensing and parallel imports (Hodgkin *et al.*, 2001; World Health Organization, 2004, 2007)
12. Financial protection of vulnerable groups	Duty toward non-discrimination and attention to the vulnerable (UN General Assembly, 1966b; UN CESCR, 2000, 2008)	Increase government funding for poor and vulnerable groups and reduce the risk of catastrophic health spending (World Health Organization, 2004, 2007)

Abbreviations: WHO, World Health Organization; AAAQ, availability, accessibility, acceptability and quality.

### Country selection

We selected 16 countries that have ratified the ICESCR, have a national health insurance system and had a low- or middle-income economy in 2015, with the exception of Chile and Uruguay (became high -income countries in 2012 (World Bank, n.d.). Our sample achieves maximum variation of WHO regions, legal families (WHO classification) and income economies (World Bank 2015 classification) (Table 2) (World Bank, n.d.; WHO, 2016).

**Table 2.** Overview of the 12 principles for access to medicines in national UHC legislation from 16 mostly LMICs

Income economy	Country	WHO region	Legal family	1. Right to health	2. State obligation	3. Transp- arency	4. Participation and consultation	5. Monitoring and evaluation	6. Account- ability and redress	7. Selection of essential medicines	8. Government financing	9. Pool user contributions	10. International assistance and cooperation	11. Efficient and cost-effective spending	12. Financial protection of vulnerable groups
Low	Rwanda	AF	CIV CUS												
	Tanzania	AF	CIV CUS												
Lower middle	Ghana	AF	COM CUS												
	Nigeria	AF	COM CUS ISL												
	Tunisia	EM	CIV ISL												
	Morocco	EM	CIV ISL												
	Jordan	EM	CIV CUS ISL												
	Indonesia	SEA	CIV CUS ISL												
	Philippines	WP	CIV COM												
Upper middle	Algeria	AF	CIV ISL												
	South Africa	AF	CIV COM												
	Turkey	EU	CIV												
	Colombia	AM	CIV												
	Mexico	AM	CIV												
High	Uruguay	AM	CIV												
	Chile	AM	CIV												

Legend: Black, strong text; grey, weak text; white, no text. Abbreviations: AF, Africa; EM, Eastern Mediterranean; SEA, South-East Asia; WP, Western Pacific; EU, Europe; AM, Americas; CIV, civil law; COM, common law; CUS, customary law; ISL; Islamic law.

### Collection of legislation

We convened multi-disciplinary country research teams (with law/medicine backgrounds) fluent in the official national language and English. Teams compiled 16 country profiles to describe the national health and legal context of access to medicines, and the relevant national laws (objectives, interrelationships with other legal instruments and text related to medicines). (The profiles are accessible here: https://figshare.com/projects/Legislating_for_universal_access_to_medicines_A_rights-based_cross-national_comparison_of_UHC_laws_in_16_countries/35054.)

First, teams identified relevant national laws by searching national government websites, online databases of national law and policy, reference lists in related academic and policy publications and cross-references in national legislation to other relevant laws. Second, teams mapped the relationship between laws, and cross-referenced our list with academic publications and governmental or other reports to verify that our collection was complete and current. Third, laws were selected for analysis if they were relevant for access to medicines for vulnerable groups and if they addressed at least one principle in the assessment tool. Laws governing the regulation, control and marketing of pharmaceuticals, or private health insurance were excluded. Fourth, at least one pharmaceutical policy expert from each country except Nigeria and Algeria reviewed our list of legislation for relevance and currency.

Most legal texts were extracted from the legislation first by one research team member, followed by one author who verified and supplemented the initial text selection. Selected texts were translated to English and, where possible, peer-reviewed by a second team member.

### Data analysis

Two authors each independently coded the strength of each principle in the legal texts on a three-point coding matrix (i.e. strong, weak or absent text) defined in the detailed methodology (see Supplementary Appendix). Generally, strong text includes a clear State commitment to a principle and an action (i.e. to adhere to the concept of essential medicines and introduce a national selection committee) and where possible related to medicines affordability and financing. Weak text includes vague commitments. Coders discussed disagreements until consensus was reached. Pharmaceutical policy experts (explained above) were invited to provide written comments on our preliminary results; minor changes were made to some codes due to more recent laws or differences of interpretation.

We reported the most and least frequent principles in national UHC law. For each principle we described the different approaches in different countries.

We also examined a relationship between the principles and national level of economic development by converting the legal recognition of each principle to a binary score: strong text or weak/absent text and compared with national economic development using the Fischer’s Exact Test (performed with SPSS version 25 with significance set at *P* = 0.05).

## Results

We included 86 domestic laws from 16 countries, ranging from two laws per country (Nigeria and Ghana) to 10 laws per country (Colombia) (see list in the Supplementary Appendix: https://dx.doi.org/10.6084/m9.figshare.6537452).

The strength of the 12 principles in UHC legislation is shown by country in Table 2. Legislation with innovative ideas is listed for each of the 12 principles in Table 3 (see full text examples in the Supplementary Appendix: https://dx.doi.org/10.6084/m9.figshare.6538403).

**Table 3.** Innovative ideas for medicines affordability and financing in national UHC legislation

**1. Right to health including essential medicines**
Indonesia: Law No. 36/2009 (2009)
Mexico: General Health Law (2017)
**2. State duty to provide pharmaceuticals**
Philippines: National Health Insurance Act (2013)
Mexico: General Health Law (2017)
**3. Transparency of governments’ action and outcomes for medicines affordability**
Philippines: Republic Act No. 7581 (1992), Republic Act No. 9502 (2008)
Chile: Law No. 20584 (2012)
**4. Participation and consultation for medicines affordability**
Colombia: Law No. 100 (1993)
Chile: Law No. 20850 (2015)
**5. Monitoring and evaluation for medicines affordability**
Philippines: Republic Act No. 9502 (2008)
Mexico: Regulations of the General Health Law in the matter of social protection in health (2014)
**6. Accountability and redress for medicines affordability**
Turkey: Patient Rights Regulation (2016)
South Africa: National Health Amendment Act No. 12 (2013)
**7. Selection of essential medicines**
Ghana: National Health Insurance Act No. 852 (2012)
Indonesia: Law No. 40/2004 (2004), Law No. 36/2009 (2009)
Uruguay: Law No. 18.211 (2007), Decree No. 265/006 (2006)
**8. Sufficient government financing for essential medicines**
Rwanda: Law No. 03/2015 (2015)
Philippines: National Health Insurance Act (2013)
Nigeria: National Health Act No. 8 (2004)
**9. Pooling user contributions for essential medicines**
Ghana: National Health Insurance Act No. 852 (2012)
Morocco: Law 65‐00 (2002), Decree No. 2‐08‐177 (2008)
Philippines: National Health Insurance Act (2013)
Turkey: Law No. 5510 (2006)
**10. International assistance and technical cooperations for medicines affordability**
Nigeria: National Health Act No. 8 (2004)
Mexico: Internal Regulations of the Health Secretariat of 19 January 2004
**11. Efficient and cost-effective spending on essential medicines**
Philippines: National Health Insurance Act (2013), Republic Act No. 7581 (1992), Republic Act No. 9502 (2008)
Indonesia: Regulation No. 28/2014 (2014), Law No. 36/2009 (2009)
**12. Financial protection of the poor and vulnerable**
Chile: Ministerial Decree No. 1 (2006), Law No. 19966 (2004), Law No. 20850 (2015)
Colombia: Law No. 1751 (2015)
Jordan: Civil Health Insurance of 2016, Decision of Council of Ministers No. 5157 on 13/8/2014, Instructions (to include pregnant women in civil health insurance) No. 9 (2006), Instructions No. 3 (2008)

The most common principles are pooling user contributions (*n* = 10 countries), rights (*n* = 9), accountability (*n* = 9), obligations (*n* = 8) and financial coverage for the vulnerable (*n* = 8). The least common principles are from the governance domain (monitoring, transparency, participation, *n* = 2–3) and the technical domain (international cooperation *n* = 1). Overall, UHC legislation from Colombia, Chile, Mexico and the Philippines codifies a high number of principles.

### 12 principles for access to medicines in national law

####  Right to health

Medicines are an explicit entitlement in Colombia, Mexico and Uruguay. A less specific universal right to health or services is found in Ghana, Tunisia, Chile, Indonesia and Turkey. Other national legislation did not address the right to access healthcare nor medicines, except the Philippines (right is conditional on financial contribution) and Nigeria (all are entitled to a basic minimum package of health services).

####  State obligation

Chile, Colombia, Indonesia, Mexico, the Philippines and Uruguay specify strong State obligations to pharmaceutical care. Uruguay and Chile require the State to guarantee access or ensure the availability of medicines in the national formulary for all people. The State is explicitly required to ensure access to pharmaceuticals without payment at the point of service and without discrimination (Mexico), to make essential goods affordable to all (Philippines), or to guarantee the essential medicines are equally available and affordable to the public (Indonesia). The Colombian government is responsible for respecting, protecting and ensuring the enjoyment of the fundamental right to health (including the provision of medicines) in line with the terms in General Comment No. 14.

State responsibilities are somewhat weak in Algeria, Jordan, Morocco and Tunisia. In Algeria, medicines prescribed in public health facilities are provided free-of-charge for in- and out-patients. African countries did not codify any State obligations toward health, with the notable exception of Ghana (to attain UHC) and South Africa (to provide healthcare to those without other forms of insurance, and some women and children).

####  Transparency

Transparency in pharmaceutical policy is observed in South Africa (price transparency), the Philippines (price ceilings and public disseminating of that information) and Chile (database of drug prices and medicines evaluation reports). The Philippines law has a comprehensive dissemination strategy targeting newspapers, television and posters in public markets, supermarkets and other public places.

Domestic health law most often framed transparency as information about the benefits package and procedure for accessing it (South Africa, Ghana, Philippines, Mexico, Uruguay, Colombia), the rights of patients (South Africa, Nigeria, Rwanda, Ghana, Turkey, Chile) and the complaints procedure (South Africa, Nigeria, Ghana, Colombia, Chile).

####  Participation and consultation

Patient/consumer participation in domestic pharmaceutical policy is permitted by law in Colombia (users who are trained physicians can join the Pharmacy Commission), Chile (patients may participate in the technical advisory commission recommending priority for high cost medicines) and Mexico (community participation, including to inform the authorities about medicines-related side effects and adverse reactions).

General community participation or empowerment in relation to health involve users in national consultation forums, decision-making bodies, National Health Council or Patient Rights Boards (Colombia, Mexico, Philippines, Rwanda, South Africa, Tanzania, Turkey and Uruguay). Laws require health authorities and governing boards to include geographic representatives and women (Nigeria, Ghana), workers (Algeria) or a representative of the national league for the defence of human rights (Tunisia). Jordan and Indonesia do not address the concept of participation.

####  Monitoring and evaluation

The Philippines’ Act on Universal Access and Quality Medicines is the only law in our sample to adopt a patient-centred approach to monitoring medicines prices and affordability. It requires regular surveys of sales prices of medicines and their effect on the household income of different economic groups. Other medicines-specific monitoring is mentioned in laws from Nigeria (‘good drug use’), Algeria (‘the market situation’ of medicines). Mexican legislation requires a periodic evaluation of the healthcare system related to eliminating financial and organisational barriers to accessing services and the access and supply of medicines. All countries except Turkey prescribe general monitoring of the health insurance or system.

####  Accountability and redress

The right to complain is codified in the domestic health legislation of Colombia, Chile, South Africa and Nigeria (about the manner of treatment), Turkey (in the event of infringement of patient rights), and Mexico and Rwanda (in relation to pharmacy services and code of ethics).

A detailed complaints procedure for patients who experience alleged violations of their health rights is described in legislation from South Africa, Ghana, and Indonesia. Innovative accountability mechanisms include a grievance committee at each health institution to decide on complaints (Philippines), a patient ombudsman to initiate or pursue complaints (South Africa), and patient rights units at health centres (Turkey). Most remaining countries reference a complaint or dispute settlement mechanism in health law (Nigeria, Rwanda, Tanzania, Algeria, Uruguay, Chile, Mexico). No accountability and redress mechanisms were identified in Tunisian, Moroccan or Jordanian law.

####  Selection of essential medicines

In Colombia, Ghana, Indonesia and Nigeria, the concept of essential medicines informs the pharmaceutical benefits package or the medicines provided in public centres, and their selection criteria, procedure and periodicity. Pharmaceuticals in other UHC packages are referred to as those on the national drug list (Rwanda), national formulary (Chile and Uruguay), explicit medical benefits (Mexico) and reimbursed medicines (Algeria). In Mexico, the criteria for prioritizing essential services are: the financial sustainability of the system, epidemiological profile and health needs, level of medical attention, which interventions are already covered and the principles of equity and distributive justice.

####  Government financing

Nigeria is the only country to codify the State duty to allocate funds (specifically 20% of the Basic Health Care Provision Fund) to provide essential medicines, vaccines and consumables for primary care. Colombia, Mexico, the Philippines and Turkey have a similar text but lack an explicit focus on essential medicines. In the Philippines, the government guarantees the financial viability of the health insurance program, which includes pharmaceuticals.

####  Pool user contributions

Mandatory pre-payment of UHC contributions is codified in domestic law in Colombia, Chile, Ghana, Indonesia, Jordan, Mexico, Morocco, the Philippines, Rwanda, Tunisia and Turkey. These laws require user contribution toward health insurance except for those unable to pay.

####  International assistance and technical cooperation

Mexico is the only country to embed technical cooperation with the international community for health technology assessment in law. Chilean law permits contributions from the international community to the Fund for High-Cost Diagnostics and Treatments. Colombia and Chile are also part of the Andean Agreement Decisions (called REMSAA resolutions) for medicines. Other countries engage in international cooperation and technical assistance for other health matters (Indonesia, Nigeria, Turkey, South Africa).

####  Efficient and cost-effective spending

The principle of efficient/cost-effective spending and related policy measures are codified in legislation from Colombia, Chile, Mexico, Uruguay, Indonesia, Turkey and the Philippines. Mechanisms for efficiencies include a positive list for health insurance (Jordan, Uruguay) that is based on prioritization (Chile, Mexico), exclusion criteria for medicines reimbursement (Colombia) based on health technology assessment (Indonesia, the Philippines), reference pricing (Algeria, Tunisia, Morocco), price ceilings/maximum retail prices (Rwanda, Philippines), regular pricing review (Ghana), generic promotion and/or substitution (Ghana, Algeria, Morocco, Mexico, South Africa) and the establishment of a national committee on medicines pricing (Turkey, Colombia) or a committee to study medicines prices (Algeria). Of notable mention is the Philippine [Medicines] Price Act that prohibits profiteering and permits medicines price freezes in emergency situations or excessively high prices.

####  Financial protection of vulnerable groups

The government finances universal access to basic health insurance for the impoverished in Mexico, the Philippines, Indonesia, Colombia and Chile. In Jordan, healthcare is provided free of charge to pregnant women and children, and to the poor who opt-in to insurance for a substantially reduced fee. Vulnerable groups and the impoverished are conditionally exempt from contributions for health services in Moroccan, Nigerian, Rwandan, Tanzanian, Ghanaian and Turkish law.

Care is provided free of charge in public centres in Algeria (for people in difficulty), Tunisia (preventative and general health services up to a geographic quota), South Africa (primary care to all and health services to pregnant women and children under 6 years) and Rwanda (medicines and reproductive health care).

####  Trends

We identified trends (described below), but, no significant relationships between specific principles and income economies.

## Discussion

Our study presents an assessment tool for access to medicines in national law and a cross-national snapshot of legal texts from 16 mostly LMICs. Legal rights and State obligations toward medicines are often embedded in national UHC law, while most principles for good governance are much less common. Some technical principles to implement medicines affordability and financing are frequently embedded in national UHC law (i.e. pooled user contributions and financial coverage for the vulnerable), while other principles are infrequent (i.e. sufficient government financing) to almost absent (i.e. seeking international cooperation). We also identified several trends in the legal text of countries from different levels of development (see below). To our knowledge, this is the first study to provide an in-depth qualitative analysis of legal text for access to medicines by systematically collecting and assessing domestic legislation against an assessment tool based on WHO’s policies on essential medicines and international human rights law. Our assessment tool serves as both a checklist for assessing national law and policy, and a ‘wish list’ to guide legal reform.

### Trends in legislation

The core purpose of this research was to fill a critical gap in knowledge by describing and comparing legal provisions for access to medicines. In addition to this objective, we identify three legislative trends more common (albeit not significant) in the upper-middle income countries (and those recently graduated to high income) than low and lower-middle income countries that we sampled. These relationships should be interpreted as hypotheses for further exploration in a larger sample of countries and/or more data points, as follows.

#### Trend 1: Explicit individual rights and state obligations

Affluent countries tend to codify universal entitlements and government duties. Some even include the right to free care at public health centres (Algeria, South Africa). This vague entitlement lacks clear State obligations and lines of accountability. On the other hand, less affluent countries generally refrain from guaranteeing the right to basic healthcare for all.

Some countries from all economic classes protect public health in legislation by prohibiting the denial of emergency medical treatment (Nigeria, Algeria, South Africa). This legal guarantee concerns only ad-hoc care for the immediate continuation of life; it fails to take a holistic approach to health that includes disease prevention and health promotion in the absence of illness and treatment of chronic diseases.

#### Trend 2: Clear boundaries to entitlements and obligations

Inevitable boundaries must be established for basic health packages. No government can provide universal access to all possible health interventions. Affluent countries tend to limit the scope of medicines provided by adopting the principle of essential medicines and mechanisms for their selection in UHC law. These countries also often recognize the principle of cost-effectiveness in relation to medicine selection/reimbursement and use health technology assessment (HTA) as the mechanism. HTA is an objective, transparent and predictable method for establishing the boundaries of an essential health services package. It also shapes the population’s legitimate expectations about which health interventions they are entitled to under UHC.

Conversely, less affluent countries define patients’ entitlements to health interventions based on the principle of available public resources (Nigeria, Tunisia). Despite being a recognized principle in the right to health, the concept of ‘available resources’ yields vague obligations and opaque entitlements when transplanted in national legislation. State action and rights realization are difficult to assess against these flexible standards, complicating the redress of violations.

#### Trend 3: Mechanisms for accountability and redress

Legislation in affluent countries affirms the right to hold the government accountable and outlines procedures to seek redress for rights violations. The Turkish Patient Rights Regulation entitles patients with health needs that cannot be met presently to request the objective justification of the State’s priority ranking on the basis of medical evidence. The right to question State decisions to provide some medicines but not others, and to receive a response, is the essence of accountability. South Africa has introduced a Patient Ombudsman who is responsible for investigating cases of rights violations in healthcare, based on complaints or his/her own initiative.

While less affluent countries do include some mechanisms for accountability and redress in their UHC laws, these are often limited to the (contributing) members of UHC schemes thereby excluding the general public who is not eligible for or cannot afford coverage (Ghana, Tanzania and Rwanda).

### Policy implications for WHO Member States

Our findings respond to the legitimate concerns of policy makers who hesitate to embed human rights principles in domestic law out of concern that they may trigger (further) rights-based medicines litigation. We provide a menu of principles and legal texts that, when applied together, may help to prevent such spurious claims. These texts establish health entitlements with boundaries through objective criteria (including cost effectiveness), transparent and participatory decision-making processes, and non-judicial accountability mechanisms to redress violations before having to resort to the courts. Recognizing the legal boundaries of the right to access to medicines informs patients’ reasonable expectations of their health system. It can also protect against excessive or unreasonable claims for immediate access to treatments at any cost. Starting from this basic package, governments should apply the human rights principle of progressive realization by continuously and expeditiously expanding the boundaries of access to medicines for all (Perehudoff *et al.*, 2016).

National law makers can undertake a ‘check-up’ of access to medicines using our assessment tool to identify strengths and weaknesses in existing domestic law. Our assessment tool and the example legal text can also be used as a guide for writing future legislation (Table 3). Particularly, less affluent countries may seek inspiration from the language that more affluent nations codify to develop their UHC schemes and scale-up access to medicines.

### Policy implications for WHO

WHO should develop a publicly accessible online repository of national health legislation, as echoed by other global health researchers (Attaran *et al.*, 2012). WHO should also publish technical advice for Member States legislating for access to medicines in UHC schemes, in line with the goal of WHO’s 2016–30 Medicines & Health Products Strategic Programme (WHO, 2017). This advice can use our assessment tool as a starting point and expand on the examples presented. Our examples translate some recommendations of the WHO Consultative Group on Equity and UHC for making fair choices toward UHC into provisions for domestic law (WHO Consultative Group on Equity and Universal Health Coverage, 2014). WHO’s technical advice could especially catalyse national governments to embed in their domestic legislation some of the under-addressed principles we identified (i.e. duty of government to sufficiently finance essential medicines and to seek international assistance).

Monitoring bodies (i.e. WHO, Office of the High Commissioner for Human Rights) can use our results to expand their indicator of government commitment to health rights, which is currently a right to health in constitutional or other national law (Office of the High Commissioner for Human Rights, 2012). Other sub-indicators could track specific State duties in national health legislation, such as for (1) the control of medicines prices, and (2) the sufficient financing of essential medicines for the poor and vulnerable. Embedding these sub-indicators in national law may have a more direct effect on patient-level access than governments codifying a constitutional right to health. Legalizing these State obligations can also support accountability and redress if rights are violated.

### Future research

Our study of 16 mostly LMICs does not investigate other countries making important strides toward UHC, such as Thailand, Viet Nam, and Kyrgyzstan, because we lacked the language capacity. Future research should contribute additional analyses of legislation from these and other LMICs countries with UHC.

The private sector plays a crucial role in developing, manufacturing and supplying medicines, and is increasingly active in UHC schemes as a health insurer and provider (Chapman, 2016b). Future research should continue to examine rights-based legal approaches to regulating the private sector in the context of UHC (Hallo De Wolf and Toebes, 2016; Tsevelvaanchig *et al.*, 2018).

National policies, particularly for pharmaceuticals, health and intellectual property, can instruct the development of health law or substitute it entirely by directing State policies and programming. Future research should investigate the content, implementation and impact of national policies in relation to access to medicines as part of the right to health.

The present study of UHC ‘law on the books’ does not examine the important question of how these laws are implemented in practice. In a different study, we conducted a follow-up report of eight right to health indicators of access to medicines in 194 countries (Perehudoff *et al.*, 2018). We did not find any relationship between having a constitutional law supporting essential medicines or national medicines policy and process or outcome indicators relevant for the right to health (i.e. government spending on medicines, national availability of essential medicines, childhood immunization rates). However, our analysis did not include UHC legislation, was at a high level of abstraction and had data from fewer than half of the expected data points. Therefore, we recommend that future studies elucidate how the laws in the present study are implemented through in-depth country case studies with more detailed and disaggregated sub-indicators, possibly based on the 24 indicators presented by the Lancet Commission on Essential Medicines Policies (Wirtz *et al.*, 2016). Subsequent research should also study whether the human rights principles we investigated help improve governance, implementation and health outcomes for a more equitable and universal provision of healthcare in practice (Tremper *et al.*, 2010).

### Strengths and limitations

First, although our sample is not representative of all LMICs with UHC, it includes countries from all world regions with a diversity of legal traditions and income economies. Most LMICs should be able to locate a comparable country in our sample and learn from its examples.

Second, we minimized the risk of reporting bias by working with multiple, trained research assistants fluent in the national language. We also verified our collection of legislation and preliminary findings with national experts, except Nigeria and Algeria. Our sources are more objective than similar studies that rely on interpretations of law and policy in academic literature and from key informants. Yet, our conclusions only reflect the retrievable laws and may underestimate the observed trends.

Third, to minimize the risk of incorrect translations or inconsistent interpretation, all research assistants were trained in the standard terminology and definitions of the 12 principles. Translations from French, Indonesian and Spanish were peer reviewed.

## Conclusion

This is the first study to systematically map, collect, and assess national UHC legislation for attributes related to access to essential medicines, particularly for vulnerable groups. Our research offers domestic law makers an assessment tool that is both a checklist and a wish list for legal reform for access to medicines. We present examples of legal texts from a range of mostly LMICs providing essential medicines through UHC. These examples may inspire other WHO Member States to adopt a human rights-based legal framework for universal access to medicines and sustainable development; they may also support the WHO Medicines & Health Products Strategic Programme 2016–30 to develop model legislation for medicines reimbursement (goal 7).

## Supplementary data

Project tools, case studies, and data are available at http://healthandgender.org/accesstomedicines.html. Supplementary data are available at *Health Policy and Planning* online.

## Supplementary Material

czy101_Supplementary_File
