# Supplementary material for: Legislating for universal access to medicines: a rights-based cross-national comparison of UHC laws in 16 countries
Source: Health Policy Plan. 2019 Dec 9;34(Suppl 3):iii48–57. doi: 10.1093/heapol/czy101 (PMC6910076; doi:10.1093/heapol/czy101)
Supplement: czy101_Supplementary_File [file czy101_supplementary_file.pdf]

List of all legislation included in the project
'Legislating for universal access to medicines'

Original article: *Legislating for universal access to medicines: A rights-based cross-national comparison of UHC laws in 16 health systems*

Authors: S. Katrina Perehudoff, Nikita V. Alexandrov, Hans V. Hogerzeil

Date: 14 June 2018

List of legislation that met the inclusion criteria, by country

Algeria

Law No. 83-11 of 1983. On Social Assurance.

Law No. 85-05 of 1985. On the protection and promotion of health.

Decree No. 92-07 of 1992. On Social Security.

Inter ministerial Decree No. 17 Joumada Ethania 1424 of 2003. On fixing the mission, organization and operation of a Medicines Reimbursement Committee.

Ordinance No. 03-07 of 2003. On patents.

Decree of 29 Chaoual 1427 of 2006. On fixing the list of medicines reimbursable by social security.

Ordinance No. 06-07 of 2006 amending Law. No. 85-05.

Law No. 15-02 of 2015. On mutualities.

Chile

Law No. 19.966 (2004) establishing a regime of health guarantees

Law No. 1 (2006) consolidating, coordinating, and systematising the text of Decree 2763 of 2009, and Laws No. 18.933 and 18.469

Decree No 264 (2003) & Decree No. 43 (2010) modifying Decree No. 264

Law No. 20.584 of 2012 on the rights and obligations of patients

Law No. 20.850 of 2015 creating a system for financing high cost medicines

Exento No. 31. (2015) approving the general standard of citizen participation in public health management

Exento No. 63. (2016) establishing a protocol of procedure for the provision of extrordinary aid

Colombia

Law No. 100 of 1993 on health reform

Decree 806 of 1998 regulating the essential public services of Social Security in Health

Decree No. 2085 of 2002 on data protection.

Decree No. 4966 of 2009 on the procedure for the declaration of existence of reasons of public interest in relation to compulsory licenses

Decree No. 1313 of 2010 establishing the requirements and procedures for authorising parallel imports

Law No. 1438 of 2011 on the general system of social security

Resolution 5592/2015 on updating the health benefits plan

Law No. 1751 of 2015 on the regulation of the fundamental right to health

Decree No. 780 of 2016 the Regulatory Decree of the Social and Health Sector

Resolution No. 6408 of 2016 on the modification of the POS benefits plan

Ghana

National Health Insurance Act No. 852 of 2012

Public Health Act No. 851 of 2012

Indonesia

Law No. 40/2004 on the National Social Security System.

Law No. 36/2009 on Health.

Law No. 24/2011 on the Social Insurance Administration Organization.

Government Regulation No. 101/2012 on Beneficiaries of Government Subsidy.

Presidential Regulation No. 76/2012 on Government Patent Implementation for Antivirals and Antiretrovirals.

Presidential Decree No. 19/2016 and Presidential Decree No. 28/2016 on Health Insurance.

Ministry of Health Regulation No. 28/2014 on the Implementation Guideline for the National Health Insurance Program.

Jordan

Civil Health Insurance Regulation No. 83 of 2004

Public Health Law No. 47 of 2008

Social Security Law No. 1 of 2014

Directory of the Civil Health Insurance Regulation, its amendments and the instructions issued thereunder. Fourth issue, 2016

Law No. 12 of 2013 on Medicine and Pharmacy

Mexico

General Health Law of 2017

Regulation in the matter of Social and Health Protection of 2011

Regulation of the General Health Law on the Provision of Health Care of 2016

Internal Regulation of the Health Secretariat of 19 January 2004

Regulations of the General Law of Health in the matter of Social Protection in Health of 2014

Morocco

Dahir No. 1-02-296 of 25 rejeb 1423 (3 October 2002) promulgating Law No. 65-00 on the code of basic medical coverage

Decree No. 2-08-177 of 28 Ramadan 1429 (29 September 2008) implementing the provisions of Book III of Act No. 65-00 relating to the medical assistance scheme

Dahir n ° 1-11-83 of 29 rejeb 1432 (2 July 2011) promulgating framework law n ° 34-09 concerning the health system and the provision of care.

Decree No. 2-11-199 of 7 chaoual 1432 (6 September 2011) amending and supplementing Decree No. 2-08-177 of 28 Ramadan 1429 (29 September 2008) implementing the provisions of Book III of Act No. 65-00 relating to the medical assistance scheme

Decree No. 2-13-852 of 14 safar 1435 (18 December 2013) on the conditions and modalities for fixing public sales prices of medicines locally produced and imported

Nigeria

National Health Insurance Scheme Decree No. 35 of 1999.

National Health Act No. 8 of 2014.

Philippines

Presidential Decree No. 996 of 1976 on Basic immunization for infants and children

Republic Act No. 7581 of 1992 on Prices

Republic Act No. 9502 of 2008 on Universally accessible cheaper and quality medicines

Republic Act No. 10606 of 2013 on National health insurance

Rwanda

Law No. 48/2015 governing the organisation, functioning and management of health insurance schemes in Rwanda.

Law No. 03/2015 governing the organisation of the community-based health insurance.

Law No. 21/2016 of 2016 on human reproduction

Ministerial Order No. 20/39 of 2016 determining the medical services provided at each level of health facilities

Ministerial Order No. 20/37 of 2015 on the Code of Ethics for Pharmacy Profession

South Africa

National Health Act No. 61 of 2003 and the National Health Amendment Act No. 12 of 2013

Medicines and Related Substances Act 101 of 1965 with relevant amendments from Act No. 90/1997, Act No. 59/2002, Act No. 72/2008, and Act No. 14/2015

Regulations Relating to a Transparent Pricing System (adopted 20 April 2004) for Medicines and Scheduled Substances under the Medicines and Related Substances Act No. 101 of 1965

Medical Schemes Act No. 131 of 1998, amended until Act No. 12 of 2004

Tanzania

Community Health Fund Act of 2001

Tanzania Commission for AIDS Act No. 22 of 2001

The HIV and AIDS (Prevention and Control) Act No. 28 of 2008

Public Health Act No. 1 of 2009

Turkey

Law on Socialisation of Health Care No. 224 of 1961

Statutory Decree on the Organisation of the Ministry of Health of 1983 amended until 14/04/1989

Patient Rights Regulation of 1998 amended until 2016

Social Insurance and Universal Health Insurance Law No. 5510 of 2006

Decision On The Pricing Of Medicinal Products For Human Use of 2015

Decree No. KHK/663 on restructuring the Ministry of Health

Tunisia

Law No. 91-63 of 1991 concerning the organisation of public health [l'organisation sanitaire]

Decree No. 98-409 of 1998 fixing the categories of reduced hospital tariffs

Decree No. 98-1812 of 1998 fixing the procedures to grant and withdraw a free health card

Law No. 2004-71 of 2004 on the national health insurance

Ministerial Order of 15 August 2007 on generic medicines in the health insurance scheme2

Decree No. 2012-2522 of 2012 fixing the categories of reduced hospital tariffs

Decree No. 2012-2521 of 2012 fixing the conditions and categories of a free health card

Uruguay

Decree No. 265/006 of 2006 on the National Medicines Formulary

Law No. 18.211 of 2007 on the National Integrated Health System

Law No. 18.161 of 2008 on State Health Services

Law 18.335 of 2008 on the Rights and Obligations of Patients and Users of Health Services

Decree No. 465/008 of 2008 on the Programs and Catalogue of Care in the National Integrated Health System
